# Effect of silibinin capsules combined with lifestyle modification on hepatic steatosis in patients with chronic hepatitis B

**DOI:** 10.1038/s41598-020-80709-z

**Published:** 2021-01-12

**Authors:** Duo-Duo Lv, You-Juan Wang, Meng-Lan Wang, En-Qiang Chen, Ya-Chao Tao, Dong-Mei Zhang, Hong Tang

**Affiliations:** 1 Center of Infectious Diseases, West China Hospital of Sichuan University, Chengdu, 610041 People’s Republic of China; 2 Health Management Center, West China Hospital of Sichuan University, Chengdu, 610041 People’s Republic of China

**Keywords:** Hepatitis B, Non-alcoholic fatty liver disease, Hepatitis B

## Abstract

The coexistence of HBV infection and hepatic steatosis is a novel characteristic of liver disease. Silibinin capsules (SC) is a silybin-phospholipid complex containing silybin as the bioactive component, which exerts a remarkable biological effect on various liver diseases, including nonalcoholic fatty liver disease (NAFLD). The purpose of this study was to investigate (1) the prevalence of hepatic steatosis in the general population and patients with chronic hepatitis B (CHB) and (2) to evaluate the effect of SC combined with therapeutic lifestyle changes (TLC) compared with TLC alone on hepatic steatosis in patients with CHB. A total of 16,451 individuals underwent transient elastography (TE) with the control attenuation parameter (CAP) measurement, among which the prevalence of hepatic steatosis was 31.1% in patients with CHB and 42.2% in the general population. The prevalence of hepatic steatosis differed between patients with CHB and the general population at an age of 40 years or older but was similar in individuals aged 39 years or younger (p < 0.05). Furthermore, in patients with CHB presenting hepatic steatosis, the post-6-month relative reduction in CAP in the SC combined with TLC group (p = 0.001) was significantly greater than in the TLC alone group (p = 0.183). The CAP distribution of different steatosis grades (S1, S2, and S3) in the SC combined with TLC group was decreased and S0 (CAP < 248 dB/m) increased significantly, but not significant in the TLC group. Thus, SC combined with TLC may effectively improve hepatic steatosis in patients with CHB.

## Introduction

Hepatitis B virus (HBV) infection is a common cause of liver diseases. Approximately 240 million people are estimated to have a chronic infection with HBV, and 2 billion people have been diagnosed with chronic hepatitis B (CHB) worldwide^1^. Considering nonalcoholic fatty liver disease (NAFLD), a growing and spreading liver disease worldwide^2^, the estimated frequency of hepatic steatosis in patients with CHB is 27–51%^3^. For patients chronically infected with HBV, the coexistence of steatosis may accelerate disease progression to hepatic cirrhosis, hepatic decompensation, and even hepatocellular carcinoma, as well as alter the effectiveness of antiviral therapy^4^. To date, hepatic steatosis has become the most prevalent chronic liver lesion in developed countries and has recently increased significantly in Asian countries^5^. Its prevalence is increasing in association with the global increase in obesity and is currently present in 20–44% of the general population^6^. Both CHB and fatty liver are common, and many patients suffer from both conditions. However, the prevalence of hepatic steatosis in patients with CHB remains unclear.

Recently, a novel tool based on the evaluation of ultrasound attenuation using transient elastography (TE) called control attenuation parameter (CAP) has been shown to correlate well with the histopathological steatosis stage^7^. This approach enables a noninvasive and accurate assessment of the stage of steatosis in patients with fatty liver^8^. Although liver biopsy has traditionally been regarded as the gold standard for steatosis grading, it may be related to complications and reduced accuracy due to variable sampling methods^9^. CAP, an economically attractive alternative to liver biopsy, is also considered a noninvasive standard for the identification of steatosis in patients with CHB^10^. In addition, the CAP measurement has good levels of intra- and interobserver agreement, allowing for population-wide screening and disease follow-up^11^. Therefore, we used CAP measurements to assess steatosis in the general population and patients with CHB.

Whether hepatic steatosis is investigated in the general population or patients with CHB, simple steatosis is considered benign and reversible, but severe steatosis may range from asymptomatic liver simple steatosis to fatty steatohepatitis, cirrhosis and liver failure, or even hepatocellular carcinoma (HCC)^12,13^. Silymarin has been used to treat liver diseases for many years^14^. Additionally, several human clinical trials have shown that silybin may be a useful treatment for NAFLD^15,16^. Indeed, silybin has antioxidant, anti-inflammatory, and antifibrotic properties^17^. As shown in the study by Jiayin Yao et al.^18^, silybin reduces intrahepatic fat accumulation, lobular inflammation, ballooning and serum fat levels. Notably, silybin has important roles in reducing visceral fat accumulation, inducing lipolysis by promoting the transcription of the adipose triglyceride lipase (ATGL) gene and inhibiting gluconeogenesis to silence the expression of some genes involved in the aforementioned metabolic pathway^18^. However, silybin has poor absorbance and bioavailability due to its low water solubility, thereby limiting its clinical applications and therapeutic efficiency. Excitingly, the bioavailability of silybin has been significantly improved by adding solubilizing substances (particularly phospholipids) to the standard silymarin pharmaceutical product^19^. Because the effect of silybin on patients with CHB-related fatty liver is not insufficient, we aimed to further investigate the efficacy of silibinin capsules (SC) at improving hepatic steatosis in patients with CHB.

CHB is the most prevalent chronic liver disease in China, and hepatic steatosis is also highly prevalent^20^. Furthermore, the coexistence of HBV infection and hepatic steatosis is a novel characteristic of liver disease and its incidence is gradually increasing in the Chinese population. Therefore, we aimed to investigate the prevalence of hepatic steatosis measured using CAP, to clarify the factors influencing the CAP level in patients with CHB, and mainly to reveal the effect of the SC therapeutic intervention on improving steatosis.

## Results

### Characteristics of the study population and prevalence of NAFLD

The characteristics of the study population are shown in Table 1. A total of 16,451 subjects were recruited for this study; 5680 participants positive for the hepatitis B surface antigen (HBsAg), and 10,771 subjects negative for the HBsAg served as the general population. More than half of the patients were male: 71.8% males among patients with CHB and 54.4% males among the general population (p < 0.001). The mean ages of patients with CHB and participants in the general population were 42.6 ± 11.2 and 46.9 ± 10.6 years, respectively (p < 0.001). In addition to BMI and high-density lipoprotein levels (HDL-C), differences in other parameters between the two study populations were statistically significant (p < 0.05).

**Table 1:** Baseline characteristics of participants in the general population and patients with CHB.

	General population (n = 10,771)	CHB (n = 5680)	P
Age, years	46.9 ± 10.6	42.6 ± 11.2	< 0.001
Male patients, n (%)	5859 (54.40)	4073 (71.8)	< 0.001
BMI, kg/m^2^	23.87 ± 3.2	23.29 ± 3.1	0.923
Waist circumference, cm	81.76 ± 10.0	81.1 ± 13.5	0.004
Hip circumference, cm	94.73 ± 5.7	94.2 ± 24.9	0.045
ALT, U/L	21 (15–31)	34 (23–51)	< 0.001
AST, U/L	23 (19–28)	31 (25–42)	< 0.001
Fasting glucose, mmol/L	5.18 ± 1.2	5.96 ± 4.7	< 0.001
TG, mmol/L	1.65 ± 1.4	1.42 ± 2.9	< 0.001
Cholesterol, mmol/L	4.93 ± 0.9	4.36 ± 2.6	< 0.001
HDL-C, mmol/L	1.39 ± 0.4	1.43 ± 2.1	0.481
LDL-C, mmol/L	2.90 ± 0.8	2.52 ± 3.2	< 0.001
ALB, g/L	47.83 ± 2.6	46.35 ± 14.0	< 0.001
GLB, g/L	27.92 ± 3.8	30.57 ± 6.9	< 0.001
TB, μmol/L	14.21 ± 6.0	18.16 ± 12.7	< 0.001
DB, μmol/L	3.90 ± 1.7	6.12 ± 6.4	< 0.001
GGT, U/L	21 (13–38)	25 (16–44)	< 0.001
ALP, U/L	72 (60–87)	78 (63–97)	< 0.001
Platelet × 10^9^/L	191 (152–232)	120 (80–164)	< 0.001
LSM, kPa	4.60 ± 1.2	10.02 ± 8.0	< 0.001
CAP, dB/m	241.99 ± 49.5	228.29 ± 49.8	< 0.001

*BMI* body mass index, *ALT* alanine aminotransferase, *AST* aspartate aminotransferase, *TB* total bilirubin, *DB* direct bilirubin, *TG* triacylglycerol, *HDL-C* high-density lipoprotein cholesterol, *LDL-C* low-density lipoprotein cholesterol, *GGT* gamma glutamyltransferase, *ALP* alkaline phosphatase, *LSM* liver stiffness measurement, *CAP* controlled attenuation parameter.

**Figure 1:**
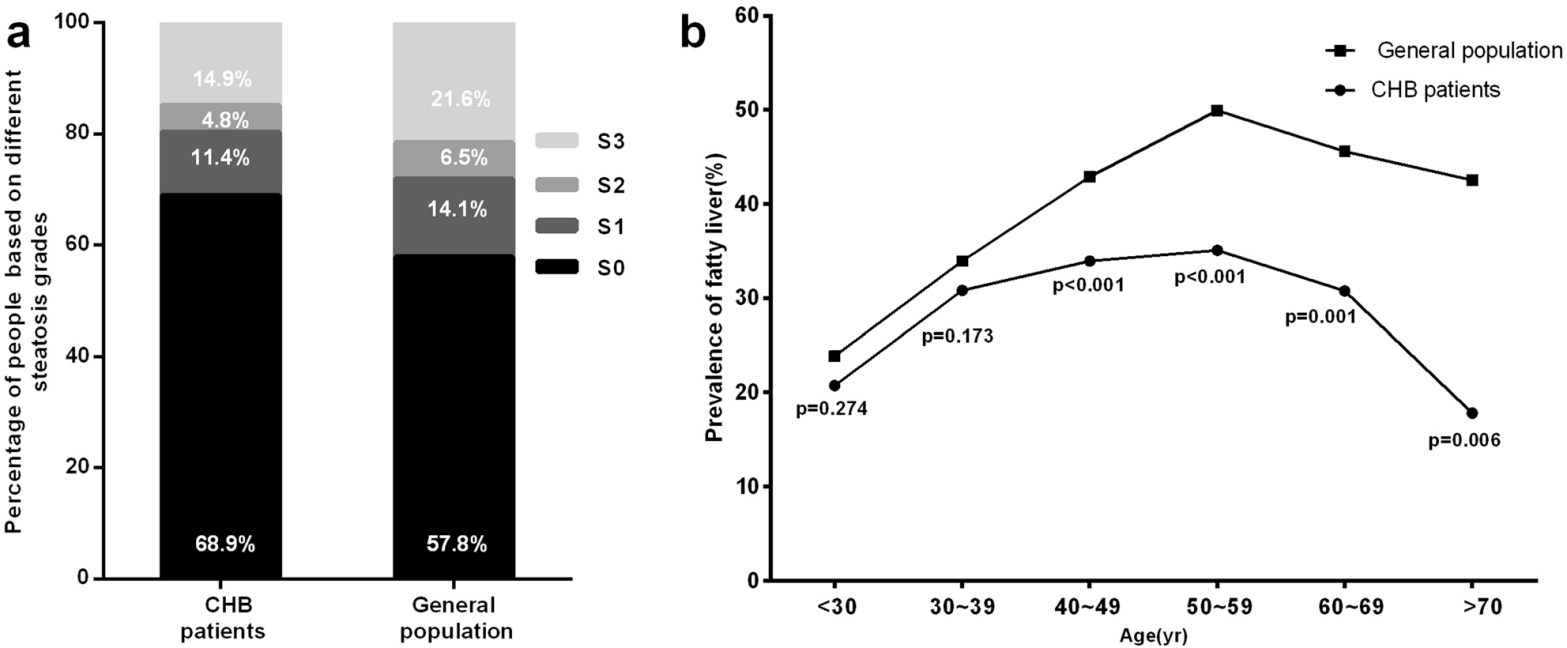
(**a**) The proportion of patients with CHB and participants in the general population with hepatic steatosis stage S0 (< 248 dB/m), S1 (248–268 dB/m), S2 (268–280 dB/m), and S3 (≥ 280 dB/m), which was determined from CAP measurement. (**b**) Prevalence of hepatic steatosis in subjects with and without hepatitis B virus infection stratified by age.

The prevalence of hepatic steatosis in patients with CHB and the general population was 31.1% and 42.2% (P < 0.001), respectively (Fig. 1). Compared to the general population, the prevalence of hepatic steatosis in patients with CHB was significantly lower. The prevalence rates of different grades of hepatic steatosis in the general population were also significantly higher than in patients with CHB (p < 0.001) (Fig. 1a). When stratified by age, patients with CHB aged 40 years or older had a significantly lower prevalence of fatty liver than participants in the general population, as shown in Fig. 1b. In both the hepatitis B and general population groups, the prevalence of fatty liver was similar in participants aged less than 40 years (Fig. 1b). Therefore, individuals with an increased CAP were significantly more commonly diagnosed in the general population than in patients with CHB, particularly among individuals aged 40–70 years.

### Factors associated with CAP in patients with CHB identified using the univariate and multivariate logistic regression analyses

A total of 1769 patients among CBH patients had CAP ≥ 248 dB/m in our study. Compared to other subjects, subjects with an elevated CAP based on TE were significantly more likely to be male; additionally, subjects with an increased CAP had a higher BMI, waist circumference, hip circumference, AST level, ALT level, triglyceride level, fasting glucose level, LDL-C level, CHOL level and platelet count, but a lower HDL-C level (p < 0.05).

In the univariate analysis, an increased CAP ≥ 248 dB/m was associated with age, the male gender; higher BMI, waist circumference, hip circumference, AST level, ALT level, triglyceride level, fasting glucose level, LDL-C level, CHOL level, DB level, GGT level, and LSM level; and a lower HDL-C level (*P* < 0.05, Table 2). A multivariate logistic regression analysis was performed to determine the factors associated with S0 compared to ≥ S1. BMI (OR 1.142, 95% CI 1.057–1.234, p = 0.001), ALT levels (OR 1.011, 95% CI 1.006–1.017, p < 0.001), TG levels (OR 1.537, 95% CI 1.255–1.881, p < 0.001), LDL-C levels (OR 1.887, 95% CI 1.172–3.034, p = 0.011) and the platelet count (OR 1.003, 95% CI 1.000–1.017, p = 0.023) remained independently associated with moderate/severe steatosis in patients with CHB (Table 2).

**Table 2:** Univariate and multivariate logistic regression analyses of factors influencing controlled attenuation parameter values in patients with CHB.

	S0 (n = 3911)	≥ S1 (n = 1769)	Univariate, OR (95% CI)	P	Multivariate, OR (95% CI)	P
Age, years	42.21 ± 11.5	43.42 ± 10.4	1.010 (1.005–1.015)	< 0.001		
Male patients, n (%)	2651 (68.8)	1422 (80.5)	1.962 (1.713–2.246)	< 0.001		
BMI, kg/m^2^	22.78 ± 2.8	24.28 ± 3.5	1.218 (1.194–1.243)	< 0.001	1.142 (1.057–1.234)	0.001
Waist circumference, cm	80.14 ± 7.9	85.56 ± 8.5	1.085 (1.077–1.094)	< 0.001		
Hip circumference, cm	92.47 ± 6.3	96.67 ± 22.8	1.099 (1.088–1.110)	< 0.001		
ALT, U/L	31 (21–47)	39 (28–57)	1.009 (1.007–1.011)	< 0.001	1.011 (1.006–1.017)	< 0.001
AST, U/L	31 (25–42)	33 (26–42)	1.001 (0.998–1.003)	0.629		
GLU	5.61 ± 1.8	5.86 ± 3.5	1.027 (0.980–1.076)	0.269		
TG, mmol/L	1.16 ± 1.0	1.63 ± 1.1	1.736 (1.526–1.975)	< 0.001	1.537 (1.255–1.881)	< 0.001
Cholesterol, mmol/L	4.21 ± 1.2	4.63 ± 4.1	1.185 (1.081–1.299)	< 0.001		
HDL-C, mmol/L	1.43 ± 0.46	1.41 ± 3.3	0.436 (0.341–0.558)	< 0.001		
LDL-C, mmol/L	2.34 ± 0.75	2.62 ± 1.1	1.522 (1.333–1.737)	< 0.001	1.887 (1.172–3.034)	0.011
ALB, g/L	45.86 ± 5.5	46.00 ± 5.1	1.005 (0.993–1.018)	0.427		
GLB, g/L	30.47 ± 6.8	30.78 ± 7.2	1.006 (0.997–1.016)	0.182		
TB, μmol/L	18.20 ± 11.9	18.09 ± 14.2	0.999 (0.994–1.005)	0.797		
DB, μmol/L	6.34 ± 7.0	5.67 ± 4.7	0.977 (0.963–0.992)	0.003		
GGT, U/L	24 (16–41)	28 (18–50)	1.003 (1.002–1.005)	< 0.001		
ALP, U/L	78( 63–97)	79 (64–97)	1.001 (0.999–1.003)	0.350		
Platelet × 10^9^/L	118 (79–162)	124 (84–167)	1.002 (1.000–1.003)	0.035	1.003 (1.000–1.017)	0.023
LSM, kPa	10.16 ± 8.3	9.70 ± 7.3	0.992 (0.985–1.000)	< 0.001		

*BMI* body mass index, *ALT* alanine aminotransferase, *AST* aspartate aminotransferase, *TB* total bilirubin, *DB* direct bilirubin, *TG* triacylglycerol, *HDL-C* high-density lipoprotein cholesterol, *LDL-C* low-density lipoprotein cholesterol, *GGT* gamma glutamyltransferase, *ALP* alkaline phosphatase, *LSM* liver stiffness measurement, *CAP* controlled attenuation parameter.

### Comparison of the effects of SC combined with TLC and TLC alone on the improvement in the CAP for fatty liver in patients with CHB

We compared the clinical parameters among the 2 groups (SC combined with TLC group vs TLC group) in Table 3. With the exception of age (42.7 ± 8.9 vs 45.49 ± 9.6 years, p = 0.004), gender (93.9% vs 80.6%, P = 0.002), ALT levels (46 (32–68) vs 34 (23–47), p < 0.001) and AST levels (37 (27–53) vs 30 (24–39), p = 0.001), statistically significant differences in demographics were not observed between the pharmacological intervention study group and the nontreatment study group. In particular, the mean value of CAP between the two groups was not statistically significantly different.

**Table 3:** Comparison of the baseline characteristics of the SC combined with TLC group and TLC group before the intervention.

	SC combined with TLC group	TLC group	P
Age, years	42.7 ± 8.9	45.49 ± 9.6	0.004
Male patients, n (%)	108 (93.9)	125 (80.6)	0.002
BMI, kg/m^2^	25.21 ± 3.2	24.2 ± 3.1	0.629
Waist circumference, cm	87.38 ± 16.1	85.2 ± 7.4	0.216
Hip circumference, cm	96.74 ± 5.6	95.12 ± 5.4	0.389
ALT, U/L	46 (32–68)	34 (23–47)	< 0.001
AST, U/L	37 (27–53)	30 (24–39)	0.001
Fasting glucose, mmol/L	5.67 ± 1.3	5.90 ± 1.9	0.881
TG, mmol/L	1.76 ± 1.2	1.55 ± 1.0	0.417
Cholesterol, mmol/L	4.18 ± 1.2	4.17 ± 0.8	0.836
HDL-C, mmol/L	1.40 ± 1.2	1.21 ± 0.4	0.546
LDL-C, mmol/L	2.43 ± 0.8	2.42 ± 0.73	0.983
ALB, g/L	45.83 ± 5.3	45.30 ± 4.1	0.281
GLB, g/L	31.37 ± 9.9	31.2 ± 6.4	0.127
TB, μmol/L	22.8 ± 38.0	19.17 ± 13.4	0.297
DB, μmol/L	9.3 ± 26.8	6.37 ± 7.1	0.815
GGT, U/L	32 (19–58)	28 (17–46)	0.117
ALP, U/L	78 (62–101)	75 (65–99)	0.811
Platelet × 10^9^/L	118 (81–169)	108 (72–168)	0.678
Undetectable HBV DNA, n (%)	90 (78.3)	122 (78.7)	0.929
HBeAg-negative, n (%)	79 (68.7)	108 (69.7)	0.469
NAs treatment, n (%)	70 (60.9)	101 (65.2)	0.449
LSM, kPa	12.31 ± 8.0	11.48 ± 8.2	0.279
CAP, dB/m	282.11 ± 37.30	272.43 ± 28.20	0.115

*BMI* body mass index, *ALT* alanine aminotransferase, *AST* aspartate aminotransferase, *TB* total bilirubin, *DB* direct bilirubin, *TG* triacylglycerol, *HDL-C* high-density lipoprotein cholesterol, *LDL-C* low-density lipoprotein cholesterol, *GGT* gamma glutamyltransferase, *ALP* alkaline phosphatase, *LSM* liver stiffness measurement, *CAP* controlled attenuation parameter.

**Figure 2:**
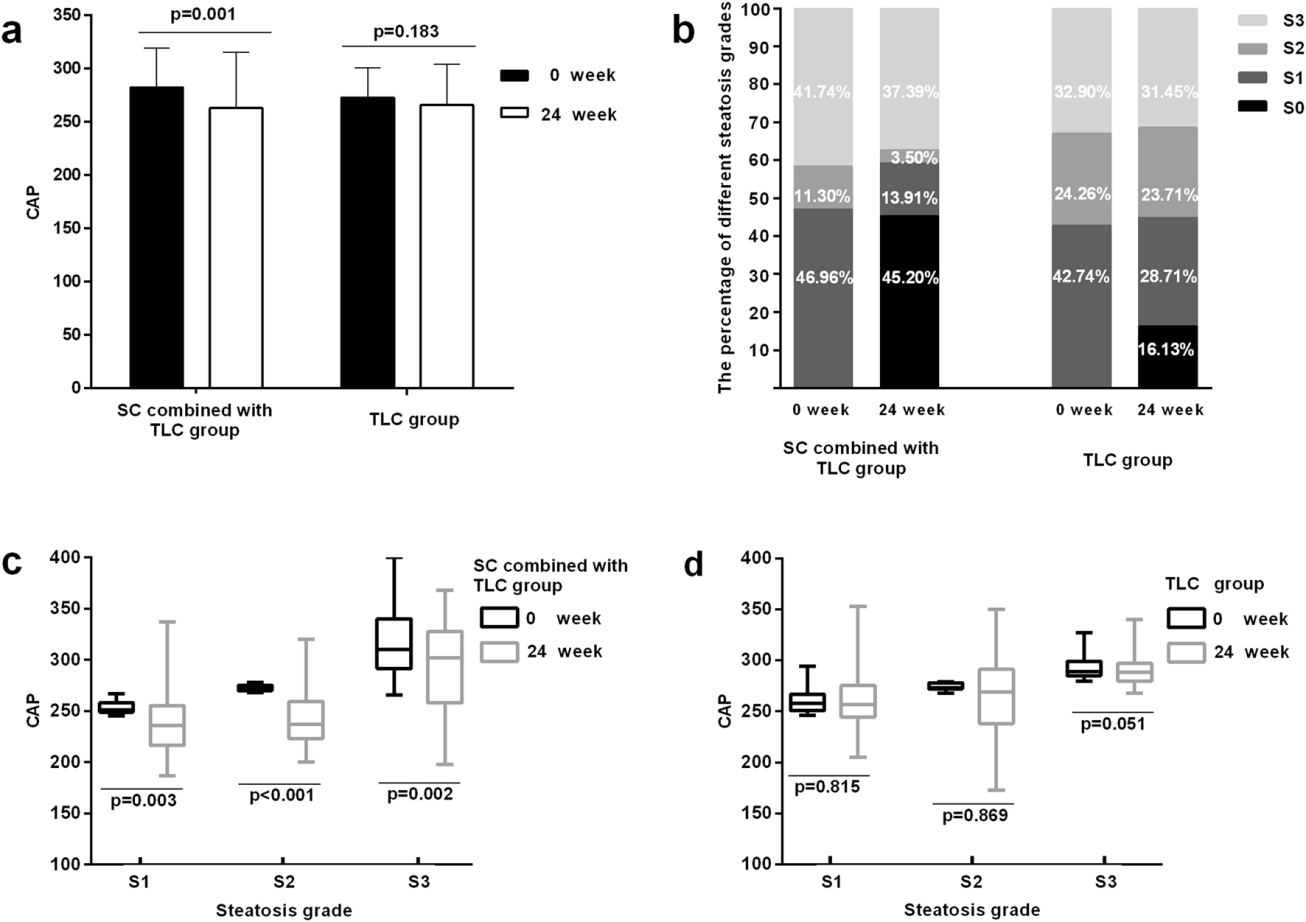
(**a**) Change in the CAP value from 0 to 24 weeks in the SC combined with TLC and TLC alone groups. (**b**) Distribution of percentages of patients with different degrees of hepatic steatosis at baseline and at the end of intervention in the SC combined with TLC and TLC alone groups, as assessed by the CAP measurement using TE. (**c**,**d**) Change in the CAP value of patients with different steatosis grades (S1, S2, and S3) from 0 to 24 weeks in the SC combined with TLC and TLC alone group.

We compared the mean changes in the CAP value at 0 week and after 24 weeks between the two study groups (Fig. 2a). Compared with 0 week, the CAP value was significantly decreased in the SC combined with TLC group after 24 weeks (282.11 ± 37.30 vs 262.93 ± 52.60, p = 0.001), and the mean CAP value in the TLC group was decreased after 24 weeks, but the difference was not statistically significant (P = 0.183). As shown in Fig. 2b, 45.20% of patients with CHB in the SC combined with TLC group showed a CAP < 248 dB/m at 24 weeks, while only 16.13% of the patients in the TLC group displayed a significantly decreased degree of hepatic steatosis, suggesting that the intervention combining SC with TLC significantly improved the degeneration of fatty liver. Similarly, reduced CAP values were observed for patients with S1 (253.6 ± 6.1 vs 235.9 ± 19.4, P = 0.003) and S2 (272.5 ± 3.0 vs 262.9 ± 25.6, P < 0.001) in the SC combined with TLC group of mild and moderate steatosis. More importantly, the CAP value for severe hepatic steatosis of stage S3 was also significantly decreased (316.7 ± 30.3 vs 290.3 ± 37.9, P = 0.002) (Fig. 2c). In contrast, TLC alone did not ameliorate the CAP values of patients with mild, moderate and severe steatosis of S1 (258.74 ± 18.9 vs 258.69 ± 25.4, P = 0.525), S2 (277.87 ± 18.13 vs 275.04 ± 21.9, P = 0.807) and S3 (292.98 ± 11.9 vs 290.78 ± 15.5, p = 0.051) after 24 weeks (Fig. 2d). A slight decrease in the CAP value for steatosis was observed in the TLC group, but no difference was observed in the patients with different steatosis grades between 0 week and after 24 weeks.

## Discussion

With the increasing pandemic of obesity, fatty liver disease has been considered one of the major causes of liver-related morbidity and mortality. In fact, the relationship between chronic HBV infection and hepatic steatosis has long been a controversial matter. Clinical studies have reported an association between HBV infection and a lower prevalence of hepatic steatosis, metabolic syndrome and hypertriglyceridemia^21^. Our study obtained consistent results; the incidence of hepatic steatosis was 31% in patients with CHB, a value that is lower than the value of 42% observed for the general population. Although the coexistence of CHB and fatty liver may reduce the incidence of hepatic steatosis, when the two diseases are present at the same time, the patient’s condition still requires our attention to monitor the progression of the disease.

Although many studies have shown that a CAP measurement obtains good results for both the diagnosis of fibrosis and the capability of assessing steatosis in patients with chronic liver disease^22^, the CAP value was strongly correlated with several metabolic parameters. According to a prospective study, the CAP value is closely associated with a higher BMI, waist circumference, cholesterol level and triglyceride level^23^. Moreover, CAP values are also significantly correlated with BMI, particularly in patients with NAFLD^24,25^. Consistent with the results of previous studies, CAP values measured in patients with CHB were strongly correlated with BMI, gender and metabolic syndrome in the present study. Therefore, the CAP value of patients with CHB appeared to be influenced by inflammation and metabolic factors, but not fibrosis. Additionally, the PLT count was an independent predictor of the CAP value in the multivariate analysis. However, a study performed by Saremi et al. did not reveal a significant difference in the mean PLT count between patients with NAFLD and healthy participants^26^. This condition may be caused by chronic hepatitis B itself. Therefore, the relationship between the PLT count and hepatic steatosis in patients with CHB requires further study.

In general, the management of fatty liver disease primarily depends on intense lifestyle changes to lose weight, but many different drugs (including incretin-based drugs, insulin sensitizers, antioxidants, lipid lowering agents, and weight loss medications) and bariatric surgery or liver transplantation have emerged, which represent therapeutic options that may be added to TLC when necessary for the management of the disease^5,27^. Notably, numerous studies have documented correlations between the therapeutic effect of Silybin on treating NAFLD with its special properties^28^. For example, according to a meta-analysis of randomized control trials conducted by Zhong et al.^29^, sylimarin may be a promising and preferred option for patients with NAFLD, particularly patients with NASH, compared with hepatoprotective drugs and drugs targeting metabolic disorder. Furthermore, Loguercio C and his colleagues found that the use of Realsil (comprised of a silybin phytosome complex coformulated with vitamin E) for 12 months significantly improved steatosis, inflammation and ballooning in HCV-positive patients with NAFLD compared with the placebo group^30^. Lifestyle interventions in terms of food intake and physical activity are a first-line approach to prevent and treat NAFLD, but these interventions are difficult to put into practice. More importantly, NAFLD encompasses various liver diseases, but most studies have focused on NASH rather than the stages of NAFLD. Here, we retrospectively studied the use of SC in patients with CHB and fatty liver. After comparing the CAP values at 0 and 24 weeks between the SC combined with TLC and TLC alone groups, the efficacy of the SC combined with TLC in reducing the hepatic fat content was very significant in the present study, as CAP values obviously decreased. Moreover, the CAP value decreased in the TLC group, but the difference was not statistically significant. Thus, SC combined with TLC may improve the CAP value for fatty liver disease in patients with CHB when combined with TLC.

This study has some limitations. First, subjects did not undergo a liver biopsy. Second, the analysis of CAP values was only conducted using a conventional M probe without using the XL probe. In addition, since we conducted a retrospective study, an assessment of adherence either to SC or to lifestyle modifications was lacking. Moreover, a limited observation duration of six months was used in the present study, and longer observation times might allow the identification of the therapeutic effects of drugs.

In conclusion, HBV infection is associated with a lower prevalence of fatty liver when the CAP measurement is used to evaluate the severity of steatosis during follow-up. Moreover, SC combined with lifestyle interventions over 24 weeks (6 months) led to effective changes in CAP values and may be more effective at reducing associated hepatic steatosis than lifestyle modifications alone.

## Methods

### Study population

**Figure 3:**
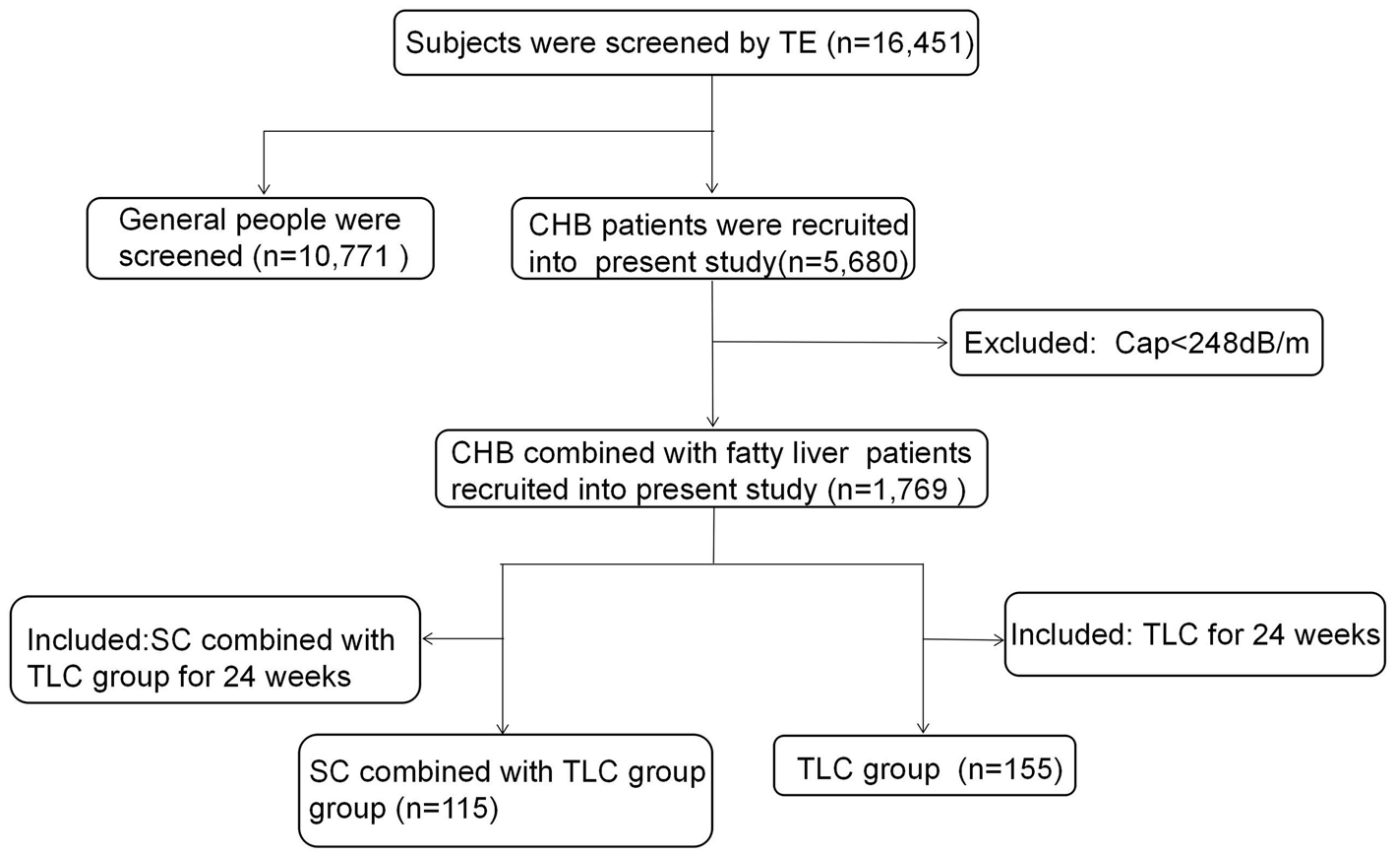
Participant disposition of the current study.

The retrospective observational study evaluated 16,451 consecutive individuals from West China Hospital who underwent a liver disease screen during the period from February 2013 to August 2017 and had a reliable liver stiffness measurement (LSM) and CAP measurement using TE (Fig. 3). In all cases, the diagnosis of CHB was based on the presence of HBsAg in serum, elevated liver transaminase levels for at least 6 months, and/or an HBV-DNA content greater than 10^4^ copies/ml^1^. Both treatment-naïve and on-treatment patients with CHB were recruited. CAP provides a standardized noninvasive measure of hepatic steatosis, and fatty liver was defined as a CAP ≥ 248 dB/m^8^. The following patients were excluded from the study: (1) coinfection with other hepatitis viruses, such as hepatitis A, C, D and E; (2) coexistence of other liver diseases, such as autoimmune hepatitis (AIH), alcoholic fatty liver disease (AFLD) and primary biliary cirrhosis (PBC); (3) decompensated cirrhosis; (4) ALT level > 5×ULN; (5) previous liver transplantation; and (6) hepatocellular carcinoma (HCC).

### CAP measurement using transient elastography

The CAP and liver stiffness measurements were obtained through TE performed by experienced operators using a FibroScan502 Touch instrument (Echosens, Paris, France). All patients underwent TE using the FibroScan M probe on the right lobe of the liver through the intercostal spaces with the patient lying in the dorsal decubitus position and the right arm in maximal abduction after fasting for at least 8 h. CAP was only calculated when the LS value was valid to ensure accurate attenuation^31^. Reliable LS and CAP values were defined using the following three criteria: (a) at least 10 valid shots, (b) a success rate (SR: the ratio of valid shots to the total number of shots) of at least 60%, and (c) an interquartile range (IQR) of less than 30% of the median LS value (IQR/M\30%). The TE results of the device estimate liver steatosis in decibels per meter (dB/m) and liver stiffness in kilopascals (kPa) ranging from 100 to 400 dB/m and 2.5 to 75 kPa, respectively^32^. Hepatic steatosis was defined as a CAP measurement of ≥ 248 dB/m, the optimal cutoff of controlled attenuation parameter for diagnosing moderate liver steatosis (S ≥ 2) was 268 dB/m, and a CAP measurement ≥ 280 dB/m was defined as severe steatosis (S3)^8^. Cirrhosis was defined as a liver stiffness > 12 kPa with a normal ALT level or > 13.4 kPa in patients with ALT levels 1–5 × the upper limit of normal^33^.

### Intervention strategy

Among the 5680 CHB patients diagnosed with TE, 115 patients who took SC capsules 70 mg^3^ times/day, orally and combined with TLC [regular diet coupled to physical activity (daily moderate exercise for 30 min-1 h)] to ameliorate fatty liver were included in the SC combined with TLC group. Likewise, 155 untreated patients with CHB-associated fatty liver were selected as the control group and received TLC alone. The observation time for this treatment was 24 weeks. No other hepatoprotective drugs were used during the treatment period.

### Clinical data

On the same day as the CAP measurement of TE was performed, demographic data (such as gender, age, waist circumference, hip circumference and body mass index) and clinical laboratory parameters (including biochemical parameters and routine blood tests) were recorded. The patients who began to receive SC in the outpatient department were regarded as the baseline time point, and demographic and laboratory data and the LSM and CAP values of participants were also collected at baseline and 24 weeks. All analyses of participants’ blood samples were conducted at West China Hospital of Sichuan University.

### Statistical analysis

Descriptive statistics of the clinical and demographic parameters are presented as the means ± SD for continuous variables, as the medians (interquartile ranges) for variables with a nonnormal distribution, and as counts and percentages for categorical variables. Demographics were compared between the general population and patients with CHB using the Student's t-test and Mann–Whitney test for continuous variables and the Chi-square test for categorical variables. A univariate analysis was performed to identify the factors influencing the CAP values of patients with CHB, after which a multivariate logistic regression model using the factors with a value of p < 0.05 in the univariate analysis was performed. Chi-square or Mann–Whitney tests were utilized as appropriate to compare continuous data between the SC combined with TLC group and TLC alone group. A p value < 0.05 was considered a statistically significant difference. SPSS 22.0 software was used to analyze the data, and figures were drawn with GraphPad Prism 6 software.

### Statement on ethical approval and informed consent

This study was approved by the West China Hospital Ethics Committee, Sichuan University. Due to the retrospective nature of the study, an informed consent waiver was approved by the Ethics Committee. All methods described in the present study were performed in accordance with the relevant guidelines and regulations.

## Data Availability

All data generated or analyzed during this study are included in this published article.
